# *Galleria mellonella* as an infection model for the virulent *Mycobacterium tuberculosis* H37Rv

**DOI:** 10.1080/21505594.2022.2119657

**Published:** 2022-09-11

**Authors:** Masanori Asai, Yanwen Li, John Spiropoulos, William Cooley, David J. Everest, Sharon L. Kendall, Carlos Martín, Brian D. Robertson, Paul R. Langford, Sandra M. Newton

**Affiliations:** a Section of Paediatric Infectious Diseases, Department of Infectious Disease, Imperial College London, London, UK; b Department of Pathology, Animal and Plant Health Agency, Addlestone, UK; c Centre for Emerging, Endemic and Exotic Diseases, Pathobiology and Population Sciences, Royal Veterinary College, Hartfield, UK; d Department of Microbiology, Facultad de Medicina Universidad de Zaragoza, Zaragoza, Spain; e MRC Centre for Molecular Bacteriology and Infection, Department of Infectious Disease, Imperial College London, London, UK

**Keywords:** Tuberculosis, *Mycobacterium tuberculosis*, *Galleria mellonella*, infection model, innate immunity, mycobacteria

## Abstract

Tuberculosis (TB), caused by *Mycobacterium tuberculosis* (*MTB*), is a leading cause of infectious disease mortality. Animal infection models have contributed substantially to our understanding of TB, yet their biological and non-biological limitations are a research bottleneck. There is a need for more ethically acceptable, economical, and reproducible TB infection models capable of mimicking key aspects of disease. Here, we demonstrate and present a basic description of how *Galleria mellonella* (the greater wax moth, *Gm*) larvae can be used as a low cost, rapid, and ethically more acceptable model for TB research. This is the first study to infect *Gm* with the fully virulent *MTB* H37Rv, the most widely used strain in research. Infection of *Gm* with *MTB* resulted in a symptomatic lethal infection, the virulence of which differed from both attenuated *Mycobacterium bovis* BCG and auxotrophic *MTB* strains. The *Gm-MTB* model can also be used for anti-TB drug screening, although CFU enumeration from *Gm* is necessary for confirmation of mycobacterial load reducing activity of the tested compound. Furthermore, comparative virulence of *MTB* isogenic mutants can be determined in *Gm*. However, comparison of mutant phenotypes in *Gm* against conventional models must consider the limitations of innate immunity. Our findings indicate that *Gm* will be a practical, valuable, and advantageous additional model to be used alongside existing models to advance tuberculosis research.

## Introduction

Tuberculosis (TB) is caused by *Mycobacterium tuberculosis* (*MTB*) [1]. Conventional animal models of TB have both biological (capacity to mimic aspects of disease, e.g. induction of granulomas) and non-biological (acquisition and maintenance cost, animal housing, and ethical restrictions) limitations that are bottlenecks in research [2]. While alternative and ethically more acceptable infection models such as zebrafish [3] and fruit flies [4] are available, these models require the use of surrogate mycobacterial species, such as *Mycobacterium marinum* and may be associated with different responses to that induced by *MTB*. Thus, there is a need for alternative *MTB* models that replicate key aspects of disease, including granuloma formation, the hallmark of human TB [5].

The larvae of *Galleria mellonella* (*Gm*) are a potential infection model [6]. As a model to study infectious disease *Gm* has already been described with over 65 bacterial and fungal pathogens, with over 1500 articles registered on NCBI PubMed in the last decade alone (search terms: *Galleria mellonella* AND infection). Uptake in *Gm* as an infection model stems from a number of advantageous properties. *Gm* possess a complex innate immune system comprised of phagocytic cells (haemocytes) that function similarly to mammalian neutrophils and macrophages. Unlike zebrafish or fruit flies, *Gm* can be incubated at 37°C, and do not require specialized maintenance facilities or equipment. Infections are typically conducted via injection which allows for accurate dosing of pathogens. This is important considering that virulence in *Gm* can often vary widely with small differences (e.g. 0.5 log CFU) in infectious dose [7–10]. *Gm* are cost-effective, their short lifespan facilitates rapid acquisition of reproducible data, and ethical approval is not required. However, as a model organism in its infancy, the experimental capability of *Gm* is limited by the lack of widely accessible immunological and molecular methods/toolbox for comprehensive characterisation of the host response to infection.

Previously, we infected *Gm* with members of the *MTB* complex (MTBC), that is, *Mycobacterium bovis* BCG Montréal (BCG *lux*) [11], and a biosafety level (BSL)-2 compliant double auxotroph of *MTB* (Δ*leuD* Δ*panCD*, SAMTB *lux*) [12]. These mycobacteria induced larval mortality, were internalized by haemocytes, survived *in vivo*, induced the formation of granuloma-like structures (GLS), and the models facilitated screening for antimycobacterial compounds [12,13]. While SAMTB *lux*, unlike BCG *lux*, preserved the virulence locus RD1, both mycobacteria were equally virulent in *Gm* [14]. This raised concerns that *Gm* could not differentiate the virulence between the members of the MTBC and/or their isogenic mutants. Additionally, feedback from the TB community indicated concerns about the relevance of relatively high infectious doses and short-time frame used in the published studies [11–13,15,16].

To address these concerns, we report here for the first time, infection of *Gm* with the widely used virulent *MTB* strain H37Rv [17], the parental strain of SAMTB *lux* [12,18]. H37Rv originates from the clinical isolate H37, isolated from a TB patient at the Trudeau Institute in 1905 [19]. H37 has evolved into two strains, the avirulent H37Ra and the virulent H37Rv [20], with the latter adopted as a reference strain due to its phenotypic similarities with the tubercule bacilli described by Robert Koch [17]. Here, we present a revised infection model utilizing a lower infectious dose and extended study length that differentiates mycobacterial virulence in the order of H37Rv > SAMTB *lux* > BCG *lux*; forms GLS in response to H37Rv, can be used as a screen for anti-TB drugs, and as a virulence screen for isogenic *MTB* mutants.

## Results

### Infection with H37Rv leads to bacterial proliferation and is more lethal for *G. mellonella* compared to BCG *lux* and SAMTB *lux*

**Figure 1.**
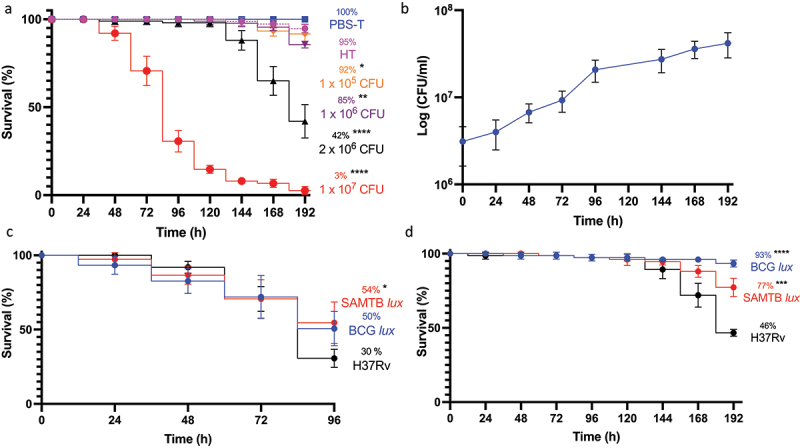
Virulence and growth of H37Rv in *Gm*. (a) Survival assay of *Gm* (n = 25, per group) challenged with varying infectious doses of H37Rv. Larval survival was recorded every 24 h for 192 h. Mock infected (PBS-T) larvae were included as controls. (b) *In vivo* survival of H37Rv (2 x 10^6^ CFU) in *Gm* (n = > 25) was measured over a 192 h time-course. CFU were enumerated from the *Gm* homogenate (n = 4, per time-point) to measure changes in the *in vivo* mycobacterial load over the course of infection. (c and d) survival assay of *Gm* (n = 25, per group) using varying CFU doses of H37Rv, BCG *lux* and SAMTB *lux*, were conducted to determine differences in mycobacterial virulence between the three strains, measured as changes in larval survival. (c) Larval survival was recorded every 24 h for 96 h (1 x 10^7^ CFU for BCG *lux* and H37Rv and 2 × 10^7^ CFU for SAMTB *lux*) or (d) 192 h (2 x 10^6^ CFU). All infected larvae were maintained in the dark at 37 °C following infection. All plotted data are the means of three (or four [Figures 1a - 2 × 10^6^ CFU]) independent experiments, and the error bars represent the SD of the means. Sample size per experiment group were n = 25. Percentage represents final larval survival. The Mantle-Cox Log-rank test with Bonferroni’s correction, (a) carried out against the mock treated (PBS-T) control or (b) respective H37Rv CFU dose, was used. * = *p* <0.05, ** = *p* <0.01, *** = *p* <0.001 and **** = *p* <0.0001.

*Gm* larvae were challenged with varying CFU doses of H37Rv to determine virulence as described [11–13,16]. Larval mortality positively correlated with increasing CFU dose (Figure 1a), where the LD_50_ (infectious dose killing 50% population over 192 h) was 2 × 10^6^ CFU. Infection with non-viable heat-treated (HT) H37Rv (2 × 10^6^ CFU) failed to induce significant larval mortality, indicating virulence required viable bacteria (Figure 1a). No adverse effects were seen in *Gm* mock-infected with phosphate buffered saline (PBS) containing 0.05% of Tween-80 (PBS-T). Bacterial infection led to symptomatic disease: melanisation (darkening of cuticle), reduced motility, and death. The incubation time prior to symptomatic disease increased as CFU dose reduced. The survival of H37Rv (2 × 10^6^ CFU) in *Gm* was evaluated by CFU enumeration from larval homogenates. H37Rv established a proliferative infection in *Gm* with 1.1 log CFUs increase over 192 h (Figure 1b). Mycobacterial load increased substantially (0.90 log CFUs) within the first 96 h post-infection (pi). Between 96 and 192 h pi, mycobacterial growth declined noticeably (0.20 log CFUs). The virulence of H37Rv was compared against BCG *lux* and SAMTB *lux* under two experimental conditions: original and revised. The “original” parameter utilized a starting inocula of 10^7^ CFU over 96 h (Figure 1c, 192 h time-course is available as **Supplementary Figure S1**). The “revised” parameter used 10^6^ CFU over 192 h (Figure 1d). The “original” parameter reflected the LD_50_ doses previously defined for *Gm* infection which were 1 × 10^7^ CFU and 2 × 10^7^ CFU, respectively, for BCG *lux* [11,13,15,16] and SAMTB *lux* [12] over 96 h. For H37Rv, 1 × 10^7^ CFU was utilized for the original parameter. For the revised model, *Gm* were infected with BCG *lux*/SAMTB *lux*/H37Rv at 2 × 10^6^ CFU. At both doses, H37Rv was more virulent than BCG *lux* (10^6^ CFU: *p* < 0.0001 and 10^7^ CFU: trend but not significant), and SAMTB *lux* (10^6^ CFU: *p* < 0.001 and 10^7^ CFU: *p* < 0.05). Significant differences in survival between BCG *lux* and SAMTB *lux* were observed at 10^6^ CFU (*p* < 0.05), but not with 10^7^ CFU. These results indicate that differences in virulence between members of the MTBC were found in *Gm* in a dose-dependent manner.

### H37Rv is internalised in haemocytes and induces the formation of GLS throughout the larval cavity

**Figure 2.**
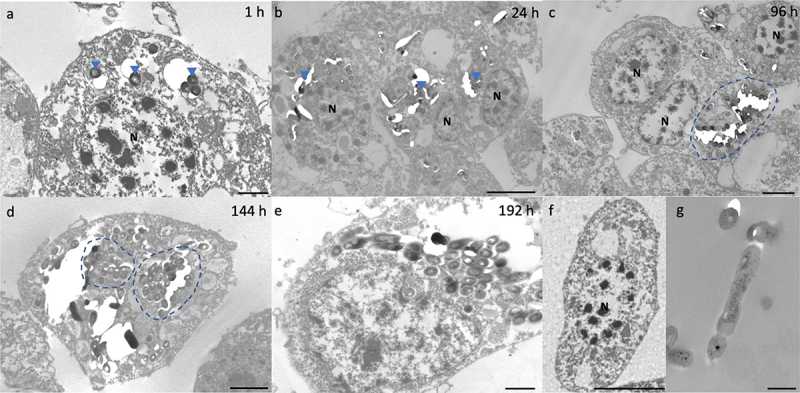
Interaction of H37Rv and *Gm* haemocytes. TEM was undertaken on haemocytes extracted from H37Rv (2 x 10^6^ CFU) infected larvae at (a) 1 h, (b) 24 h, (c) 96 h, (d) 144 h and (e) 192 h post-infection (pi). (f) Haemocytes of uninfected larvae and (g) H37Rv were used as the controls. (a) as early as 1 h pi, H37Rv bacilli (blue arrowheads) were internalised by the phagocytic haemocytes. (b) at 24 h pi, small clusters of H37Rv bacilli (blue arrowheads) were contained by aggregates of haemocytes. (c) by 96 h pi, early GLS surrounding a central mass of mycobacteria (highlighted in blue) were visible. (d-e) Abundance of intracellular bacilli (d, highlighted in blue) were observed in increasing frequency with the progression of disease. (e) by 192 h pi, haemocytes were primarily necrotic, losing cell integrity with leakage of intracellular materials and bacilli. N = nucleus. Scale bars represent a, e: 1 μm, c, d: 2 μm, b, f: 4 μm and g: 400 nm.

Internalization of H37Rv by hemocytes was visualized by transmission electron microscopy (TEM) as early as 1 h pi (Figure 2a). By 24 h pi, hemocyte aggregates containing small clusters of bacilli were apparent (Figure 2b). By 96 h pi, hemocytes surrounding a large central mass of bacilli became more common (Figure 2c). With disease progression, individual hemocytes appeared to contain larger numbers of bacilli (Figure 2d). By 192 h pi, hemocytes appear necrotic, as adjudged by loss of cell integrity and leakage of intracellular materials (Figure 2e). Intracellular bacilli were observable at all time-points, supporting the proliferative nature of the infection as determined by CFU enumeration. Intracellular H37Rv lacked any distinguishable changes in their phenotype, such as formation of intracytosolic lipid inclusions (ILIs).

**Figure 3.**
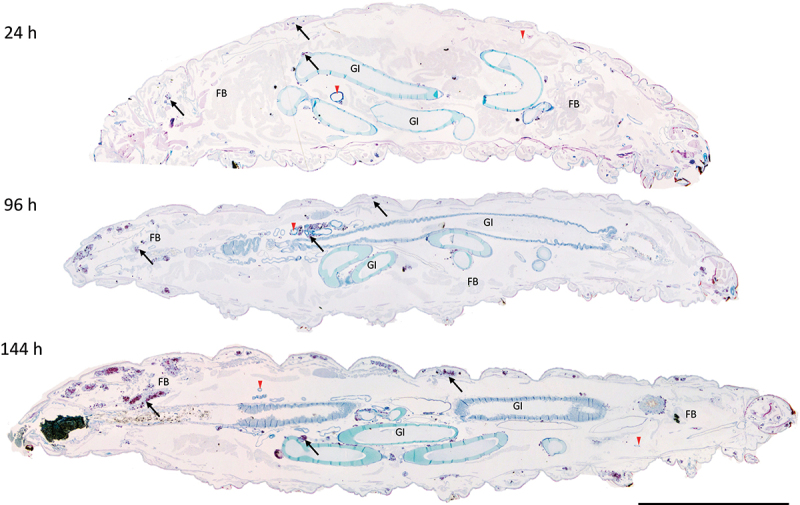
Formation of granuloma-like structures (GLS) in *Gm* infected with H37Rv. Sagittal histological sections of *Gm* infected with H37Rv (2 x 10^6^ CFU) at (a) 24 h, (b) 96 h and (c) 144 h post-infection. ZN stained sections highlights GLS (black arrows) observed in increasing abundance and size over the course of infection . FB = fat body, GI = gastrointestinal tract, and red downward triangle = trachea. Scale bar represents 1000 μm.

**Figure 4.**
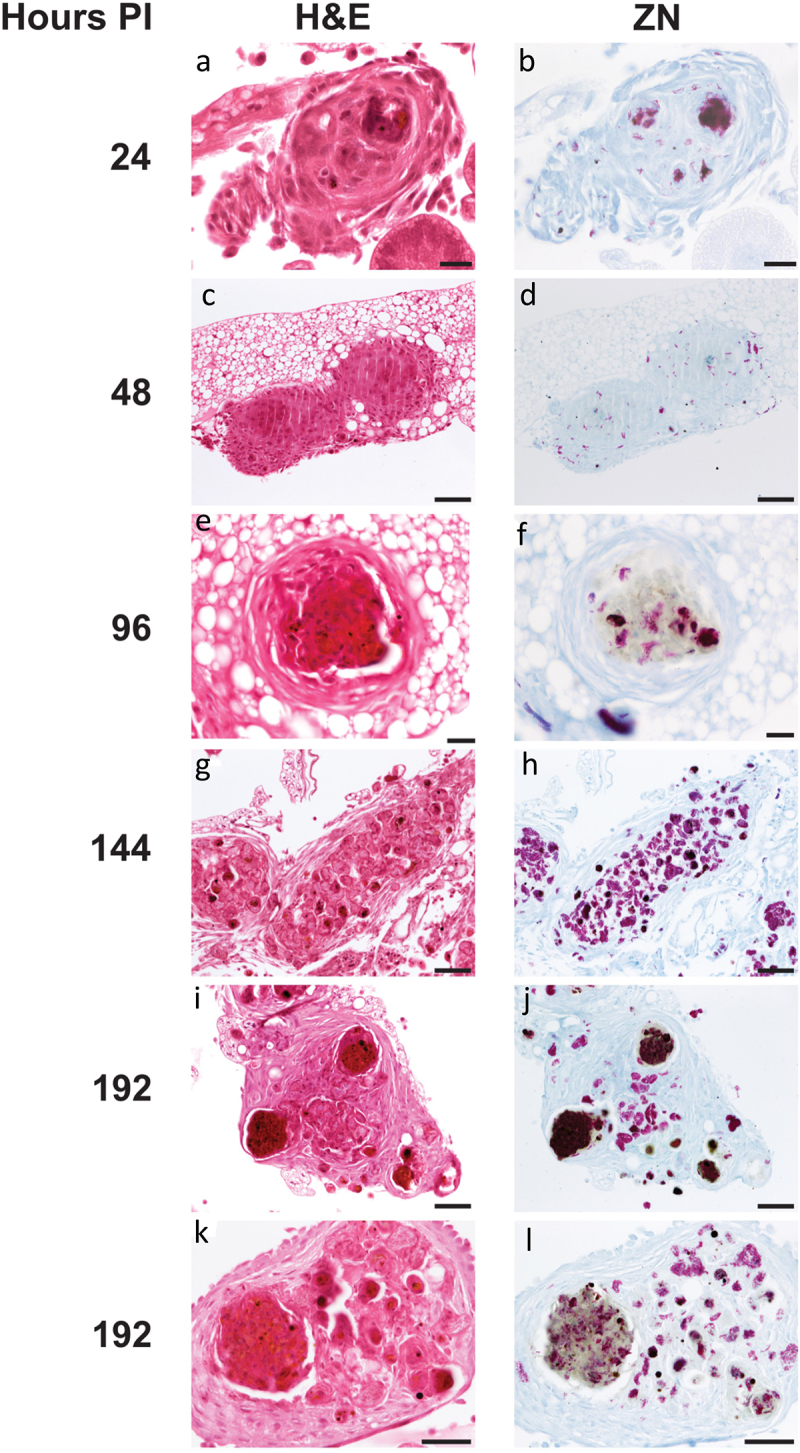
Histological analysis of *Gm*-H37Rv infection. Histological tissue sections of larvae infected with H37Rv (2 x 10^6^ CFU) were prepared and processed for H&E (a, c, e, g, i, and k) or ZN (b, d, f, h, j, and l) staining. Granuloma-like structures (GLS) were visualised at 24 h (a, b), 48 h (c, d), 96 h (e,f) 144 h (g, h) 192 h (i-l) post-infection. The complexity of host cell arrangement forming the GLS varied from (a, b) organised to (c, d) unstructured. The physical state of H37Rv bacilli contained within the GLS varied over time with mixtures of individually distinct active clusters of bacilli of varying size (bright pink) or densely packed highly ZN-reactive amorphous material released from dead/dying bacilli (dark purple). Loss in ZN affinity (grey mass) was observed in structures 96 h pi onwards (f, j, l). Cell necrosis, visible in H&E stained sections characterised by cellular fragmentation, loss/fading of nuclear staining, and decreased staining (due to appearance of pale spongy pockets) were observed in the areas associated with densely packed ZN reactive material. Scale bars represents 20 μm for a, b, e, and f; 100 μm for c, d and g-j; 50 μm for k and l.

**Figure 5.**
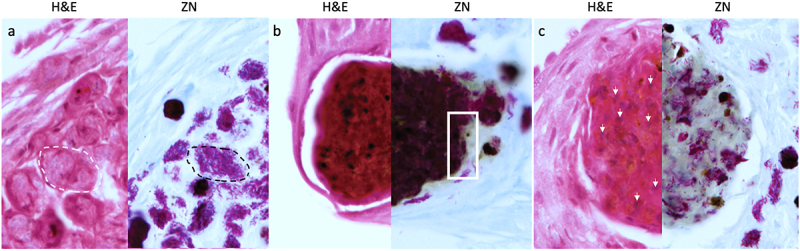
Unique varieties of granuloma-like structures (GLS) found in *Gm* infected with H37Rv. Magnified images of ZN and H&E stains presented in Figure 4, highlighting key areas of interest. (a) large aggregates of individually distinct H37Rv bacilli were observed (ZN, circumscribed by black dotted line). A GLS clearly defined as circular eosinophilic area with faded spongy pockets of staining (H&E, circumscribed by white dotted line). (b) a GLS containing dense ZN reactive material, with localised peripheral loss in ZN reactivity as indicated in the area enclosed by the black square. Foci of intense H&E staining most likely indicates host cell necrosis. (c) a GLS associated with predominant loss of ZN reactivity. In contrast to (b), non-ZN reactive masses were associated with less intense H&E staining, and host cell nuclei were more easily distinguishable (as highlighted by the white arrows); likely indicating that the level of host cell necrosis is low despite the presence of mycobacterial mass.

**Figure 6.**
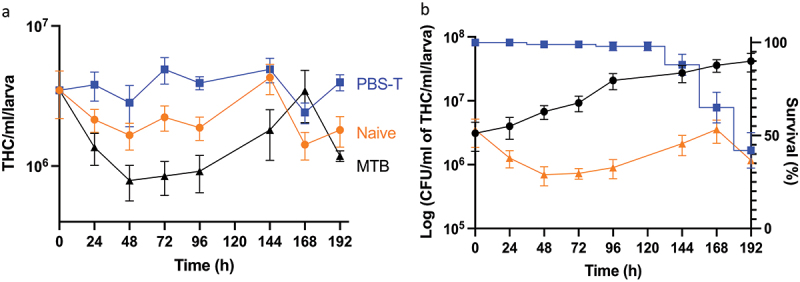
Changes in the total number of circulating haemocytes in *Gm* infected with H37Rv. (a) the total haemocyte count (THC) was measured from H37Rv (2 x 10^6^ CFU), PBS-T (mock infected), or naïve larvae (n = 4, per time-point) every 24 h over a 192 h time-course, with the exception of 120 h post-infection (pi). Plotted are the means of three independent experiments, and the error bars represent the SD of the means. (b) Changes in THC, relative to larval mortality (as presented in Figure 1a) and *in vivo* H37Rv load (as presented in Figure 1b) of infected larvae (2 x 10^6^ CFU) over the course of 192 h time-course. Suppression of THCs within the first 96- h pi, indicates diversion of circulating haemocytes into sessile state as GLS are formed. However, containment does not eliminate infection, as indicated by the proliferation of H37Rv bacilli during the corresponding time-points. By 96–192 h pi, GLS succumb to the replicating bacilli, leading to a breach in containment, inducing further immune responses as indicated by the rise in THCs back to the t = 0 h level. Nevertheless, the immune response is overloaded by the growth of H37Rv, inducing larval mortality, as indicated by the increase rate of larval mortality over the corresponding time-points.

GLS were found in increasing frequency and size during infection throughout the larval cavity as shown by Ziehl-Neelsen (ZN) staining of sagittal whole larval sections (Figures 3 and 4). These GLS comprise *Gm* cells organized around clusters or foci of H37Rv (Figure 4b). Both viable (pink ZN staining) and dead/decaying (dense purple ZN-masses lacking defined shape) bacilli were present. Corresponding sections stain intensely with Hematoxylin and Eosin (H&E) (Figure 4a), indicating host cellular necrosis. Following initial containment, mycobacteria were found in small aggregates within GLS at 48 h pi, (Figure 4d), in less complex arrangements compared to those at 24 h pi. H&E staining at 48 h indicated minimal levels of host necrosis (Figure 4c). At later timepoints, H37Rv appeared to replicate within GLS, as indicated by the abundance of pink ZN-stained bacilli (144 h pi, Figure 4h). The corresponding 144 h H&E-stained section shows *Gm* cells forming a clear border surrounding infected *Gm* cells, characterized by a circular eosinophilic area with faded spongy pockets of staining (Figure 4g and 5a). Furthermore, GLS contained melanized (brown/black) residues of probable digested mycobacterial material (Figure 4h). By 192 h pi (Figure 4j), GLS contained a variety of infection foci, including melanized, proliferative, and dead/dying bacilli. As with other structures, areas of host necrosis co-localized with mycobacteria (Figure 4i). Interestingly, at 192 h, GLS containing primarily gray non-ZN-reactive masses were apparent (Figure 4i), a similar structure was identified as early as 96 h pi (Figure 4f), and peripheral loss of ZN-reactivity was also observed (highlighted in Figure 4j and 5b). Relative to ZN-reactive foci, host cell nuclei were more clearly visualized in H&E stains of ZN negative foci (Figure 4k and 5(b,c)), indicating reduced host cell necrosis. These non-ZN-reactive foci were restricted to GLS, which were well organized. The formation of GLS, *in vivo* proliferation of bacilli and larval mortality correlated with the change in the number of circulating hemocytes found within the infected larva over the course of infection (Figure 6).

### The *G. mellonella-*H37Rv infection model can be used to determine antimycobacterial drug efficacy

**Figure 7.**
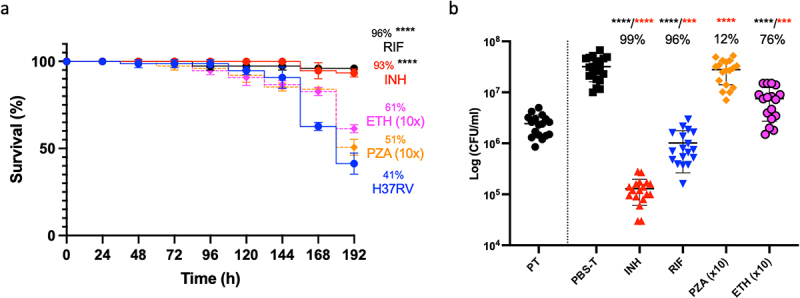
Treatment of H37Rv infected *Gm* using clinically recommended antimycobacterial compounds. Larvae (n = 25) were infected with H37Rv (2 x 10^7^ CFU) and treated using one of the following antimycobacterial compounds: INH (5 mg/kg), RIF (10 mg/kg), ETH (150 mg/kg) or PZA (250 mg/kg) using concentrations recommended for treatment of adult TB, scaled relative to body mass of the larva (200 mg). ETH and PZA were used at 10x the recommended dosage. Infection was allowed to establish for 72 h prior to treatment. Following infection, larvae were incubated in the dark at 37 °C. (a) INH or RIF treated larvae showed significant improvements in larval survival relative to the mock treated (PBS-T) controls. Larval survival was monitored every 24 h for 120 h post-treatment (or 192 h inclusive of incubation period). (b) All treatments (with the exception of PZA) led to a significant reduction in *in vivo* H37Rv burden (%), measured via CFU enumeration of H37Rv from homogenised *Gm* (n = 4, per time-point). Plotted are the means of (a) three or (b) five independent experiments, and the error bars represent the SD of the means. (a) the Mantle-Cox log-rank test with Bonferroni’s correction was carried out against the mock treated (PBS-T) controls. (b) One-way ANOVA test with Holm-šídák multiple comparisons between PBS and treatment: **** (black) = *p* <0.0001, degree of freedom = 4 and F-value = 43.79. Two tailed unpaired student’s *t*-test between PT and treatment: *** (red) = *p* <0.001 **** (red) = *p* <0.0001.

The *Gm*-H37Rv infection model was evaluated as a screen for drug efficacy with established antimycobacterials using adult TB treatment doses [21] scaled relative to the body mass of *Gm* (200 mg): isoniazid (INH, 5 mg/kg), rifampicin (RIF, 10 mg/kg), ethambutol (ETH, 15 mg/kg), and pyrazinamide (PZA, 25 mg/kg). Supplementary Table S1 lists the MICs. A single compound/dose was injected 72 h pi. INH or RIF treatment led to significant improvements (*p* < 0.0001) in larval survival relative to mock-treated (PBS-T) control (**Supplementary Figure S2**). ETH and PZA failed to improve larval survival. Treatment with ETH 10x (150 mg/kg) resulted in non-significant improvements in larval survival (61%, ETH 1x [37%]) relative to the PBS-T control (Figure 7a). No improvement in survival was observed with PZA 10x (250 mg/kg) treatment. At 120 h post-treatment there were significant reductions in H37Rv load (*p* < 0.0001) with INH, RIF, and ETH (10x) relative to the PBS-T control (Figure 7b). Only a 12% reduction in CFU load was found with PZA (10x) relative to the control. The activity of INH, RIF, and ETH was compared to mycobacterial load at the point of treatment (PT) (Figure 7b), and the CFUs recovered from the mock treated control as reference for growth and treatment activity (bactericidal or static). Relative to PT, INH, and RIF treatment led to significant reductions in mycobacterial load (*p* < 0.0001 and *p* < 0.001, respectively), indicating bactericidal activity. For ETH, while a significant level of growth (*p* < 0.001) was detected relative to PT, a significant reduction (*p* < 0.0001) in CFUs relative to the mock treated control, indicates bacteriostatic activity.

### *G. mellonella* can be used to distinguish relative virulence of H37Rv mutants

**Figure 8.**
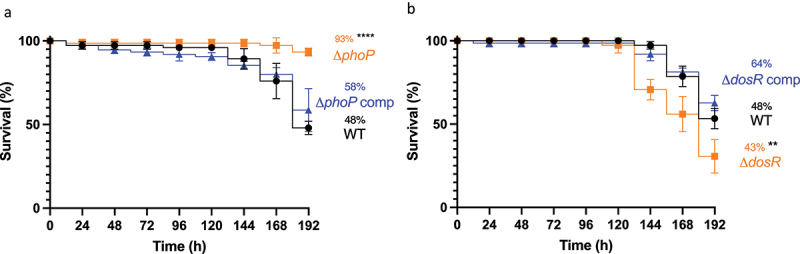
Determining virulence of isogenic mutants Δ*phoP* and Δ*dosR* against H37Rv wild-type in *Gm*. Larvae (n = 25, per group) were challenged with (a) Δ*phoP* or Δ*phoP* complement (Δ*phoP* comp), (b) Δ*dosR* or Δ*dosR* complement (Δ*dosR* comp). Larvae infected with H37Rv wild-type (WT) were utilised as the virulence control. Virulence was assessed via larval survival assay over a 192 h time-course. Infected larvae were maintained at 37 °C in the dark, and survival was recorded every 24 h. All data plotted are the mean of three independent experiments. Percentage represents final larval survival. Error bars represents the SD of the means. The Mantle-Cox log-rank test with Bonferroni’s correction was conducted against the WT. ** = *p* <0.01 and **** = *p* <0.0001.

The use of *Gm* as a screen for comparative *MTB* virulence, was evaluated using two isogenic H37Rv mutants, Δ*phoP* and Δ*dosR*, chosen based on their altered phenotype in traditional TB infection models, that is, mice [22–27], guinea pigs [28], rabbits [25] and non-human primates (NHPs) [29]. Virulence of these mutants was assessed in *Gm* at an infectious dose of 2 × 10^7^ CFU, over 192 h. Larval challenge with Δ*phoP* led to significant (*p* < 0.0001) attenuation in virulence relative to the WT, with larval survival of 93% and 48%, respectively (Figure 8a). In contrast to Δ*phoP*, larval challenge with Δ*dosR* led to significant (*p* < 0.01) potentiation of virulence relative to WT, with larval survival of 30% and 53%, respectively (Figure 8b). For both Δ*phoP* and Δ*dosR*, larval challenge with complemented strains resulted in restoration of virulence to near WT levels (statistically not significant: 58% and 62% survival, respectively). Attenuation of Δ*phoP* and hypervirulence of Δ*dosR* were in-line with results reported in a severe combined immunodeficient (SCID) mouse infection model [22,27].

## Discussion

We have previously published BSL-2 compliant BCG *lux* [11] and SAMTB *lux* [12] infection models. However, subsequent TB community feedback raised concerns that use of surrogate strains was not equivalent to virulent *MTB*, and the incubation period was too short. Therefore, we have characterized a *Gm-MTB* infection model using fully virulent H37Rv [17], and compared this to our original BCG *lux* [11] and SAMTB *lux* [12] infection models with original (10^7^ CFU) and revised (10^6^ CFU) infectious doses, and an extended incubation period (96 h to 192 h). *Gm*-H37Rv infection with these revised infection parameters, resulted in proliferative infection similar to SAMTB *lux* [12]. The revised parameters better reflect the chronic nature of TB, as the period prior to symptomatic disease is longer (+96 h relative to 10^7^ CFU dose). Whilst this incubation period is shorter and the infectious dose is higher than those used in traditional mammalian models, it is commensurate with the short life span of *Gm* and with the advantages of the low cost, low maintenance, and speed.

Comparison of H37Rv, BCG *lux* and SAMTB *lux* virulence in *Gm*, with both original and revised infection parameters, found that mycobacterial virulence could be differentiated, but with some limitations. With the original parameters, the ability to differentiate virulence between H37Rv and BCG *lux* or SAMTB *lux* was limited, as no significant change in virulence was found between H37Rv and BCG *lux*. However, with revised infection parameters the comparative virulence order was H37Rv > SAMTB *lux* > BCG *lux*. Previously, we hypothesized that the high abundance of *Gm* leucine and pantothenate [30,31] allowed SAMTB *lux* to bypass the double auxotrophy for optimal growth [12]. However, comparison with the H37Rv data, suggests that the double auxotrophy influences growth and virulence, although the mechanism is unknown. The use of other mycobacteria, such as *Mycobacterium smegmatis* and other non-tuberculous mycobacteria (NTM) for comparison, were considered. However, such comparison would be uninterpretable at the dosage and incubation period of this study, as the rate of multiplicity in NTMs are substantially higher than in the MTBC [32]. Moreover, *M. smegmatis* (5 x 10^3^ CFU) has already been studied in *Gm*, resulting in 80% larval survival after 17 days of incubation [33]. Furthermore, wide variety of NTMs have been screened, with all models having the capacity to differentiate between virulent and avirulent strains [10].

The use of commercially available *Gm* prevents for a lengthy study, as majority of non-lethal dose and control larvae begins to pupate and metamorphose into adult moth between 96 and 192 h. Our current institutional guidelines prohibit the study of *Gm* beyond the larval stage, as adult moth are an environmental pest and flight risk adds to biohazardous challenges. Furthermore, while the larval stage does not require ethics approval, the pupal stage does and requires a separate pest licence for up keep. Based on our previous *Gm*-BCG *lux* proteomics study, there is a need for innate immune induction to inhibit larval pupation, and that involves the activity of the insect metalloproteinase inhibitor (IMPI) protein, which is known to regulate metamorphism [15]. It is likely that lower doses of MTBC, unlike NTMs, do not inhibit pupation during our study duration, in part because of differences in rates of multiplication. Therefore, for study beyond the 192 h, researchers could utilize early instar larvae with longer lead time to pupation. However, such work requires rearing of an in-house *Gm* colony and feeding of infected larvae to ensure growth to last instar, both requiring additional time, resources, and facilities, thereby removing major advantages of this model.

In immune-competent animal models with adaptive immunity, infection is regulated over time [34–36]. However, lacking adaptive immunity, *Gm* relies on its innate immune response, controlling the rate of mycobacterial replication through cellular responses including phagocytosis and the formation of GLS (also known as nodulation or encapsulation) [37]. These cellular responses are supported by the humoral response producing antimicrobial effectors such as antimicrobial peptides (AMPs), reactive species, phenolic compounds, and antimicrobial enzymes (e.g. lysozyme) [15,38,39]. Evidence that the *Gm* immune response kills mycobacteria is seen by histological staining of dead/necrotic H37Rv. However, the response is insufficient to control infection, and without additional input from adaptive immunity, *Gm* eventually succumbs to proliferative bacilli, which are disseminated from the GLS [12]. This narrative is supported by changes in total hemocyte count (THC) in the circulation during the infection. During the non-symptomatic incubation period, hemocytes are recruited from the hemocoel to form GLS [40,41], lowering the THC, implying active maintenance of GLS to contain the bacilli. Over time, GLS are overwhelmed, and bacilli are released (96–168 h pi) inducing symptomatic disease. Dissemination of active bacilli from GLS into the extracellular environment stimulates larval immune response to increase the abundance of circulating hemocytes. Ultimately, immunity succumbs to the uncontrolled replication of bacilli and initiates an unstoppable cascade of humoral responses, leading to host death. Current study of the TB disease is limited to the acute phase of infection; represented by our study length and the uncontrolled mycobacterial replication in GLS, dissemination of active bacilli and phagocytosis by hemocytes to develop additional GLS. However, while GLS are formed, it is unknown whether they represent those granulomas found in animals and humans, especially in LTBI.

Previously, in the *Gm*-BCG *lux* model, ILIs were found in intracellular bacilli [11], and in mycobacteria recovered from granuloma-associated foamy macrophages [42]. Their formation is triggered by intracellular stress, and are a crucial energy source for survival during non-replicative infection [43]. Such response by BCG *lux* in *Gm* was previously reported with the detection of mycobacterial diacylglycerol O-acyltransferase in the hemolymph [15], presence of which is classically associated with ILI formation [43]. There was no ILI formation in intracellular H37Rv or intracellular SAMTB *lux* [12], perhaps reflecting the proliferative nature of the infection. The lack of ILIs in H37Rv and SAMTB *lux* may be attributable to the RD1 locus, which BCG *lux* lacks [14]. RD1 encodes the ESX-1 secretory system and 6 kDa early secretory antigenic target (ESAT-6), 10 kDa culture filtrate protein (CFP-10), required to escape the intracellular environment [44]. Lacking RD1, BCG *lux* may preferentially induce the formation of ILIs as a stress response against oxidative and hypoxic stress, minimizing damage from the intracellular environment, and maximizing its chance of survival [45]. Based on our observations in the BCG *lux* study, some mycobacteria have the potential to enter a persistent like state, as evident by ILI formation. However, its significance within the *Gm* model remains unclear, whether they areeventually degraded by the host or reactivated.

While our TEM data confirmed the internalization of mycobacteria by hemocytes, the identity of the receptor required to initiate mycobacterial phagocytosis is unknown and will remain so until the necessary tools to investigate such questions are developed for *Gm*. Only two of six hemocyte sub-types, plasmatocytes and granulocytes, are believed to be phagocytic [37]. However, owing to the lack of markers required to differentiate the sub-types via fluorescence microscopy or flow cytometry, there are no accurate and reproducible methods enabling differentiation. While the phagocytic activity of hemocytes has been published [46–50], studies have assumed that all adherent cells are phagocytic and that non-adherent cells are non-phagocytic, which skews the data. This is evident from hemocyte comparison against neutrophils and macrophages, which reported reduced phagocytic uptake in hemocyte relative to their mammalian counterparts, where assays have not considered that not all adherent cells may be phagocytic [46,51]. Once phagocytosed, intracellular mycobacteria are likely exposed to the oxidative burst driven by NADPH oxidase homologs found in *Gm* [51], which includes the induction of neutrophil extracellular trap (NET)-like structures known as insect hemocyte extracellular traps (IHET) [52], which were visualized in aggregates of *ex vivo* hemocytes recovered from *MTB* infected *Gm* (**Supplementary Data 3)**. In summary, a comprehensive analysis of the role of the oxidative burst, and the individual role of phagocytic/non-phagocytic hemocytes in *MTB* defence, requires both markers and methods to differentiate hemocytes and specific innate markers, none of which are currently available.

Histological analysis of *Gm* during H37Rv infection revealed containment of bacilli (both actively replicating and dead/decaying) in GLS of increased frequency, size, and complexity compared to the original model [12]. GLS are a generic and non-specific cellular response of *Gm* to isolate non-host material. While the mechanisms are unknown, the formation of GLS is driven by plasmatocytes and granulocytes, sub-types of hemocytes, through a cellular process known as nodulation [37,38]. Encapsulation may follow to contain the primary nodule in a capsule [37]. These multi-cellular defence structures have been reported in other *Gm*-bacteria models (e.g. *Mycobacterium abscessus* [33], *Escherichia coli* [53] and *Clostridium perfringens* [54]), and in *Gm*-fungal (e.g. *Candida albican*s [41], *Aspergillus fumigatus* [55]) models. While non-specific, GLS formation is useful to model granuloma-inducing diseases, such as those caused by the MTBC or *Madurella mycetomatis* [56,57]. As the innate immune system of *Gm* can differentiate pathogens and selectively synthesize appropriate AMPs [38], it is likely that GLS have a pathogen-specific composition. This will be testable when suitable reagents (e.g. antibodies and molecular markers) are available. Furthermore, a study into the roles of the remaining four hemocytes: prohaemocytes, spherulocytes, oenocytoids, and coagulocytes in innate immune response; and a more comprehensive understanding of how plasmatocytes and granulocytes forms GLS are required for a full characterization of host biology [58,59].

Some *Gm*-H37Rv GLS, contained non-ZN-reactive foci. While previously reported in the SAMTB *lux* model [12], H37Rv infection led to more of such foci. The emergence of non-ZN-reactive foci occurred at all stages of infection. Classically, loss of ZN-reactivity is associated with non-replicative bacilli [60], triggered by a switch in phenotype from active to non-replicating [60]. Under stress, mycobacteria shorten the lipid chains within the mycolic cell wall [61], and do not retain ZN primary dye (carbol fuchsin) [61]. Typically, this is associated with other phenotypic changes, for example, accumulation of ILIs. However, the intracellular H37Rv in *Gm* lacked ILIs. We hypothesize that H37Rv infection of *Gm* results in both active and non-replicating mycobacterial populations. Future studies to determine the feasibility of *Gm* as an infection model for non-replicating *MTB* will characterize these non-ZN-reactive populations using combinations of auramine-O, polyclonal antimycobacterial antibody, and lipophilic Nile red staining [60,62,63].

The *Gm*-H37Rv infection model was evaluated for antimycobacterial drug screening using INH, RIF, ETH, and PZA. To address concerns that drug administration 1 h pi may preferentially treat extracellular mycobacteria, the period prior to treatment was extended to 72 h pi, with CFU enumeration at 120 h pi compared to our previous studies with SAMTB *lux* and BCG *lux* [12,13]. CFU enumeration rather than luminescence (which measures metabolic activity) was used as the measure of antimycobacterial activity, because of concern that drug activity can affect metabolic activity and result in inaccurate efficacy data [64].

All treatments, except for PZA, showed significant improvement in larval survival and/or reduction of *in vivo* H37Rv mycobacterial load. Changes in treatment parameters had no effect on the efficacy of INH, RIF, ETH, and PZA compared to those reported in the SAMTB *lux* and BCG *lux* drug screening assays [12,13]. RIF and INH were the most efficacious, with activities comparable to use in C3HeB/FeJ and C3H mouse models [65,66]. ETH was efficacious only at 10x the recommended clinical dosage, and our observations align with bacteriostatic activity reported in BALB/C mouse models [67]. However, as high concentrations of ETH typically induce bactericidal and not bacteriostatic activity [68], our observations suggest host degradation of ETH or a suboptimal physiological condition for full drug potency. This also aligns with the lack of significant improvement in larval survival outcome following treatment. PZA lacked substantial antimycobacterial activity at all dosages. PZA/POA targets the pantothenate biosynthesis pathway by inhibiting PanD (aspartate decarboxylase) [69]. Inhibition of pantothenate biosynthesis disrupts critical metabolic functions (such as fatty acid and ATP synthesis) and is bactericidal [69]. However, the abundance of pantothenate (32.8 mg/kg) in *Gm* may allow H37Rv to bypass this PZA inhibition [30]. The discrepancy between survival and reduction in mycobacterial burden likely originates from the hypothesized modulation of mycobacterial PDIM by PZA/POA [61]. We acknowledge that drug treatment may enhance survival but not necessarily reduce the mycobacterial load in an appropriate manner (as evident with ETH and PZA). Therefore, we recommend that future studies conduct both time-kill and CFU enumeration to minimize risk of generating false-positive data.

Comparison of mycobacterial isogenic mutants in animal models is widely used to determine whether a gene of interest encodes a virulence or pathogenicity factor. Here, we show that the *Gm* model can also differentiate *MTB* Δ*phoP* and Δ*dosR* mutants and complemented strains. Deletion of *phoP* from the PhoPR two-component regulatory system [24,70] prevents the secretion of ESAT-6 by ESX-1 [71], functionally attenuating virulence. Δ*phoP* was attenuated in *Gm* compared to WT, which was reversible by complementation. The Δ*phoP* attenuation in *Gm* was comparable to that of BCG *lux*, and that found in SCID mice [22].

*dosR* plays a role in modulating mycobacterial metabolism in the transition from active to non-replicative state, following exposure to intracellular stress [72,73]. Δ*dosR* was more virulent in *Gm* relative to WT in terms of larval death; an effect reversible by complementation. Our results contrast with the majority of Δ*dosR* screening conducted in NHPs, C57BL/6, BALB/C, and C3HeB/FeJ mice, guinea pigs, and rabbits [25,26,28,29], which reported avirulent or indifferent outcomes. However, our results agree with those in SCID mice, which utilized the same Δ*dosR* strain used in this study [27]. Different infection outcomes are likely due to the lack of a *Gm* adaptive immune response. In immune-competent DBA mice, the H37Rv Δ*dosR* mycobacterial load was 8-10-fold higher than WT during the acute stage of infection [27], but returned to the WT level after induction of the adaptive immune response. Therefore, as both *Gm* and SCID mice lack functional adaptive immune responses, both hosts succumb to infection during the acute stage of infection.

Our results indicate that *Gm* can be used to identify *MTB* virulence genes. However, as highlighted by Δ*dosR*, the results should be interpreted with caution in light of the constraints of the model. Future studies with other mycobacterial mutants known to be attenuated in mammalian models will establish the utility of the method.

This study has highlighted the basic interactions of *Gm* and H37Rv and the data presented should serve as a strong foundation for the adoption and validation amongst the wider TB researching community. While further understanding in the *Gm* immunological responses, such as detection of inflammatory markers (e.g. IL-1β and TNF-α), release of necrotic markers (HMGB1) and identification of phagocytic receptors on the hemocytes are needed, we lack the scientific tools required for such studies to take place. This lack of a toolbox is an Achilles' heel for *Gm* and one that is widely recognized, and is an area of research that is actively undergoing development to further push the boundaries of this model [74].

In summary, this study highlights the viability of *Gm* as a host for virulent *MTB*, capable of replicating a key aspect of TB infection (e.g. GLS). However, the significance of GLS and its relevance to granulomas observed in other *in vivo* models remains to be determined. We have demonstrated that using different infection parameters *Gm* can differentiate between members of the MTBC in terms of virulence. We have also shown that the *Gm-MTB* model can be used to screen antimycobacterial drugs, and compare isogenic mutants. In both cases, the results were comparable to those reported in traditional infection models.

## Methods

### Mycobacterial strain and growth conditions

Mycobacterial strains utilized in this study are listed in Table 1. Liquid cultures were Middlebrook 7H9 broth (BD, USA), supplemented with 10% albumin dextrose catalase (BD, USA), 0.2% glycerol (Sigma-Aldrich, UK), and 0.05% Tween-80 (Sigma-Aldrich, UK). For solid cultures, mycobacteria were grown on Middlebrook 7H11 agar, supplemented with 10% oleic albumin dextrose catalase (BD, USA) and 0.5% glycerol. Liquid cultures were grown in an orbital shaker at 37°C at 220 rpm to mid-log phase optical density (OD)_600_ 0.6–0.8 (1 × 10^8^ CFU/ml). For growth on agar, plates were incubated in a static incubator at 37°C with 5% carbon dioxide for 3 weeks.

**Table 1.** List of mycobacterial strains utilized in this study.

Mycobacteria	Additional supplement for selection	Source
*MTB* H37Rv TMC 102/ATCC 35837 (H37Rv)	None	ATCC, USA
*MTB* H37Rv Δ*leuD* Δ*panCD* pMV306hsp+*lux* (SAMTB *lux*)	Hygromycin*(50 µg/ml) Kanamycin^(20 µg/ml) Leucine^(25 mg/ml) Pantothenate^(24 µg/ml)	Prof. William Jacobs Jr. (Albert Einstein College of Medicine, USA)
*M. bovis* BCG Montréal vaccine pSMT1 (BCG *lux*)	Hygromycin*(50 µg/ml)	Prof. Douglas Young (Imperial College London, UK)
*MTB* H37Rv Δ*phoP*	Hygromycin*(50 µg/ml)	(70)
*MTB* H37Rv Δ*phoP* complement	Hygromycin*(50 µg/ml) Kanamycin^ (20 µg/ml)	(70)
*MTB* H37Rv Δ*dosR*	None	(27)
*MTB* H37Rv Δ*dosR* complement	None	(27)

List of mycobacterial strains utilised, the additional supplements required for growth (* Roche Diagnostics, USA; ^ Sigma-Aldrich, UK) and the source of strains.

### Acquisition and maintenance of *G. mellonella*

Last (6^th^) instar *Gm* larvae were purchased from Livefoods Direct (Sheffield, UK). Healthy larvae were selected based on color (cream lacking melanization), mass (200–250 mg), size (2–3 cm), and a high level of motility. Dead (non-responsive to physical stimulation) or melanized larvae were discarded. Healthy larvae were stored in vented plastic containers (with wood chippings), in the dark at 18°C. Larvae were stored for no more than one week and were not fed at any point.

### *G. mellonella* infection with H37Rv, BCG *lux*, and SAMTB *lux*

For infection, mid-log phase culture were pelleted at 3000 g for 10 min. Pellets were washed twice in PBS-T (PBS [Sigma-Aldrich, UK] containing 0.05% of Tween-80 [Sigma-Aldrich, UK]). Mycobacteria were adjusted to the desired CFU inocula using OD_600_ measurements of 0.6, 1.5, 8, and 12 as a relative measure for 1 × 10^8^, 2 × 10^8^, 1 × 10^9^, and 2 × 10^9^ CFU/ml, respectively. For HT inocula, viable cultures (2 × 10^8^ CFU/ml) were incubated in a heated (80°C) water bath for 1 h. In all experiments, inocula were plated out for CFU to validate the infectious dose.

Larval infection was carried out as described [11–13,15,16]. Prior to infection *Gm* were acclimatised to room temperature for 2 h and topically decontaminated using 70% ethanol. *Gm* injection was undertaken on a disposable injection platform of filter paper (for absorption of any leaked haemolymph), taped onto a Petri dish to create a raised platform. Larvae were placed onto the injection platform on their backs and secured using tweezers to expose the pro-legs. Larvae were injected with 10 μl of mycobacterial suspension via the last-left pro-leg using a micro-syringe (25-gauge, SGE Analytical Science, Australia). Infected larvae were transferred from the platform to a Petri dish lined filter paper and incubated in a portable CULTURA mini incubator (Sigma-Aldrich, UK) inside a Class 1 microbiological safety cabinet (MSC), in the dark at 37°C, for the duration of experimentation.

### Preparation of antimycobacterial compounds and treatment of H37Rv infected *G. mellonella*

Antimycobacterial compounds were purchased from Sigma-Aldrich, UK. For treatment of infected larvae, first-line compounds were prepared according to manufacturer’s guidelines for the treatment of adult TB [21], or based on prior *Gm*-MTBC treatment studies [12,13], relative to the body mass of *Gm* (200 mg): INH (5 mg/kg), RIF (10 mg/kg), ETH (15 or 150 mg/kg), and PZA (25 or 250 mg/kg). Treatment of H37Rv infected (2 × 10^6^ CFU) larvae were conducted as described [12,13] with modifications. Treatment was given as a 10 μl injection via the last right pro-leg, 72 h pi. Treated larvae were incubated for 120 h post-treatment inside a Class 1 MSC in the dark at 37 °C

### *G. mellonella* survival assay for evaluation of mycobacterial virulence and treatment efficacy

For determination of mycobacterial virulence, larvae were infected, and monitored every 24 h over a 192 h time-course (unless otherwise stated), as described [11–13,15,16]. Infected larvae were considered dead when they failed to respond to physical stimulation. Pupated larvae (the next stage of the *Gm* lifecycle) were discarded and recorded as having survived. Larval survival was similarly recorded for treatment efficacy and presented as a Kaplan-Meier survival curve, comprised of data generated from three independent experiments unless otherwise stated.

### Measurement of *in vivo* H37Rv survival in *G. mellonella*

Changes to the bacterial load during infection or following treatment were determined via CFU enumeration. At each time-point, four larvae were randomly selected and individually homogenized in a lysing matrix tube containing six 1/8-inch metal beads in 800 μl of PBS-T, using a FastPrep F120 (MP Biomedicals, USA) at 6.0 m/s for 1 min. For CFU enumeration, serial ten-fold dilutions were plated on Middlebrook 7H11 agar, supplemented with 20 μg/ml of piperacillin (PIP, Sigma-Aldrich, UK) to inhibit the growth of native *Gm* flora [11]. PIP has no inhibitory activity on H37Rv (MIC = 320 μg/ml).

### Total haemocyte count (THC)

At each time-point, four larvae were bled by piercing the area between the head and the thorax with a 30 gauge needle, and 40 μl from each larva pooled into a 1.5 ml reaction tube containing ice-cold insect physiological saline (IPS) [75]. IPS maintains near physiological conditions, inhibiting coagulation and/or melanization. Hemolymph mixtures were pelleted (800 g for 10 min), hemocytes were carefully resuspended in ice-cold IPS. Ten microliter of cell suspensions were loaded into a disposable counting chamber (VWR, UK), and counted using a light microscope. THCs of naïve and PBS-T mock infected larvae were controls. THCs were derived from three independent experiments.

### *TEM and histological analysis of G. mellonella-*H37Rv *interaction*

*Gm* were infected with H37Rv (2 × 10^6^ CFU) and processed for TEM and histological analysis as described [11,12]. In brief, for TEM, hemocytes of 10 larvae were collected at each time-point and washed as described for THC. Pelleted hemocytes were resuspended in 3% glutaraldehyde, fixed for 10 min, pelleted (800 g for 10 min), and stored at 4°C. Pelleted hemocytes were treated with 1% osmium tetroxide, dehydrated in ethanol and embedded in resin. Sliced sections (70–90 nm) were mounted, stained with uranyl acetate (0.5%) and lead citrate (3%), and examined using an Tecnai bioTWIN transmission electron microscope (FEI Company, USA). Healthy hemocytes from naïve larvae and suspensions of H37Rv were used as controls. For histological analysis, three larvae were fixed at each time-point by injecting 100 μl of 10% buffered formalin. Fixed larvae were cut into halves along the dorsal line. Larvae were processed for histology using the Sakura Tissue-Tek VIP (Sakura, USA), embedded in paraffin wax using the Histostar™ Embedding Center (Fischer Scientific, USA), and sliced into 4 μm sections using an RM2135 microtome (Leica Biosystems, Germany). Sections were mounted onto glass slides and processed for H&E or ZN staining and examined using an Eclipse 80i light microscope (Nikon, Japan). Uninfected larvae were fixed and used as controls.

## Statistical analysis

All the data were analyzed and plotted using Prism 9 (GraphPad Software Inc, USA). Where appropriate, the Mantle-Cox log rank test with Bonferroni’s correction, one-way ANOVA with Holm-Šídák multiple comparisons or two-tailed unpaired *t*-test with multiple comparison correction was used.

## Supplementary Material

Supplemental Material

## Data Availability

The data supporting the findings of this study are available from figshare with 10.6084/m9.figshare.19668768, under open licence (CC BY 4.0).
