# Supplementary material for: Galleria mellonella as an infection model for the virulent Mycobacterium tuberculosis H37Rv
Source: Virulence. 2022 Sep 11;13(1):1543–57. doi: 10.1080/21505594.2022.2119657 (PMC9481108; doi:10.1080/21505594.2022.2119657)
Supplement: Supplemental Material [file KVIR_A_2119657_SM2614.zip › supplementary/Supplementary Figure 1.pdf]

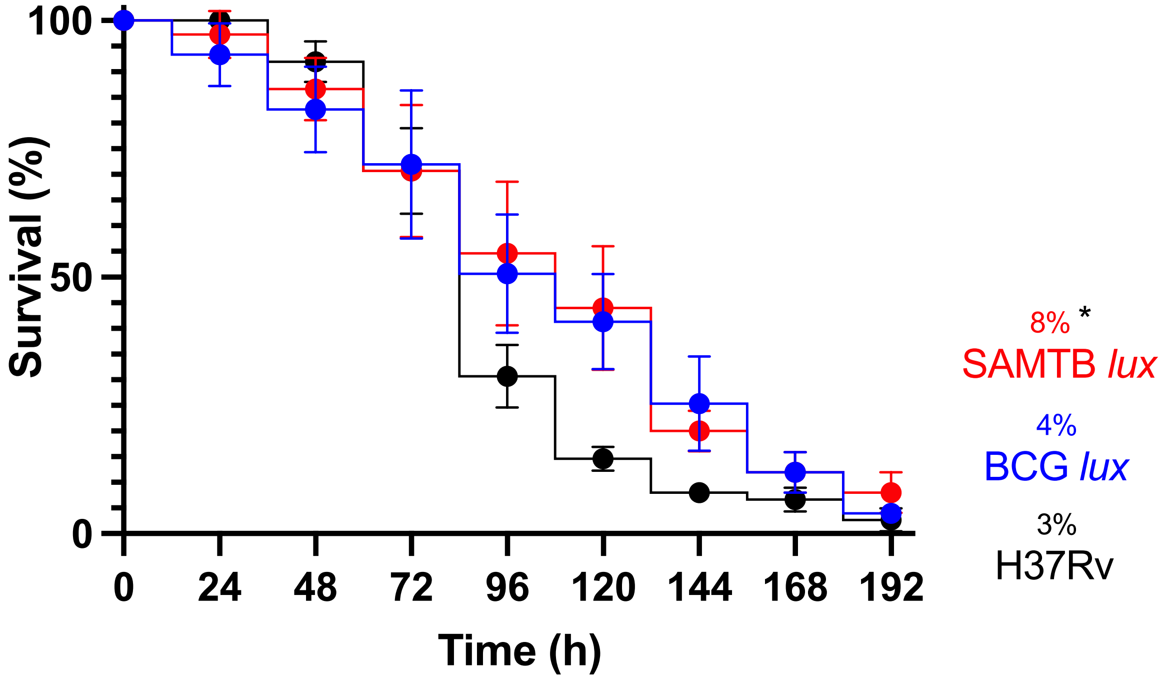

Supplementary Figure 1: *Gm* survival following infection with H37Rv, BCG *lux* or SAMTB *lux*. Survival assay of *Gm* (n = 25, per group) larvae challenged with 10^7 CFU of H37Rv, BCG *lux* or SAMTB, were conducted to determine differences in mycobacterial virulence between the three strains, measured as changes in larval survival over 192 h. The Mantle-Cox log-rank test with Bonferroni's correction was conducted against the WT. * = $p < 0.05$.
