# Supplementary material for: Galleria mellonella as an infection model for the virulent Mycobacterium tuberculosis H37Rv
Source: Virulence. 2022 Sep 11;13(1):1543–57. doi: 10.1080/21505594.2022.2119657 (PMC9481108; doi:10.1080/21505594.2022.2119657)
Supplement: Supplemental Material [file KVIR_A_2119657_SM2614.zip › supplementary/Supplementary Figure 2.pdf]

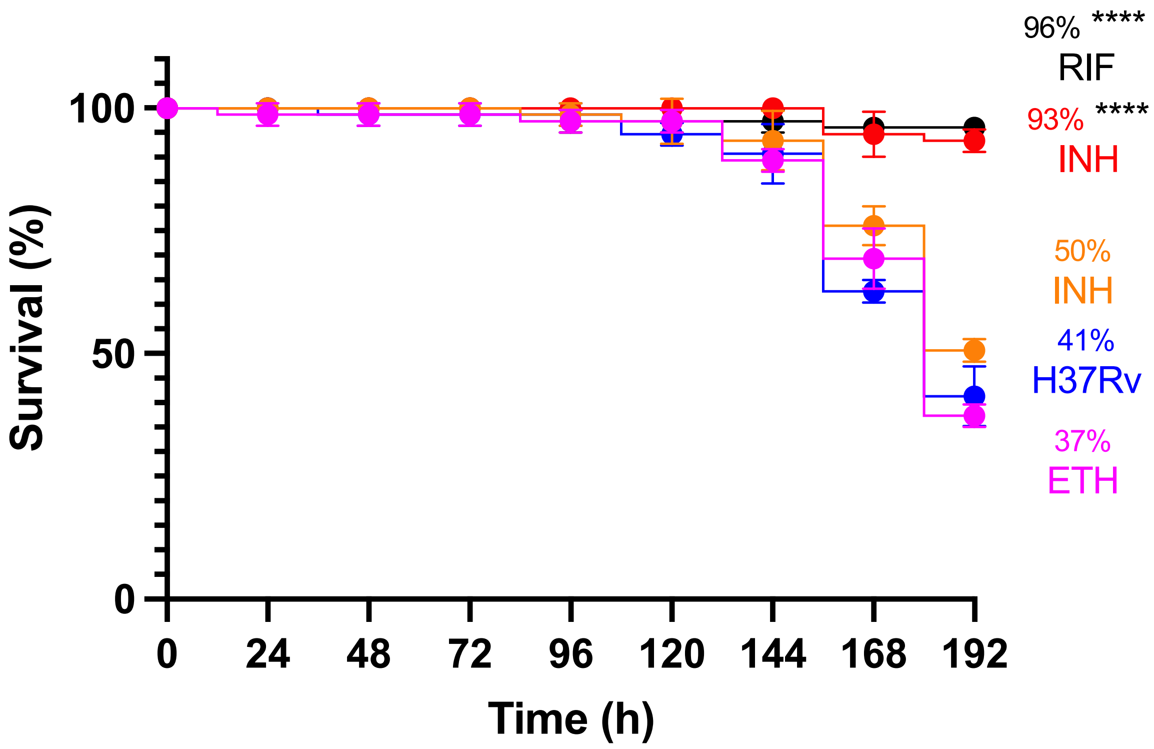

Supplementary Figure 2: Treatment of H37Rv infected *Gm* using clinically recommended antimycobacterial compounds. Larvae (n = 25) were infected with H37Rv (2 x 10^7 CFU) and treated using one of the following antimycobacterial compounds: INH (5 mg/kg), RIF (10 mg/kg), ETH (15 mg/kg) or PZA (25 mg/kg) using concentrations recommended for treatment of adult TB, scaled relative to body mass of the larva (200 mg). Infection was allowed to establish for 72 h prior to treatment. Following infection, larvae were incubated in the dark at 37 °C. Only INH and RIF treated larvae showed improvements in larval survival relative to the mock treated (PBS-T) control. Larval survival was monitored every 24 h for 120 h post-treatment (or 192 h inclusive of incubation period). Plotted are the means of three independent experiments, and the error bars represents the SD of the means. The Mantle-Cox log-rank test with Bonferroni's correction was carried out against the mock treated (PBS-T) control. **** = $p < 0.0001$.
