# Supplementary material for: Galleria mellonella as an infection model for the virulent Mycobacterium tuberculosis H37Rv
Source: Virulence. 2022 Sep 11;13(1):1543–57. doi: 10.1080/21505594.2022.2119657 (PMC9481108; doi:10.1080/21505594.2022.2119657)
Supplement: Supplemental Material [file KVIR_A_2119657_SM2614.zip › supplementary/Supplementary Figure 3.docx]

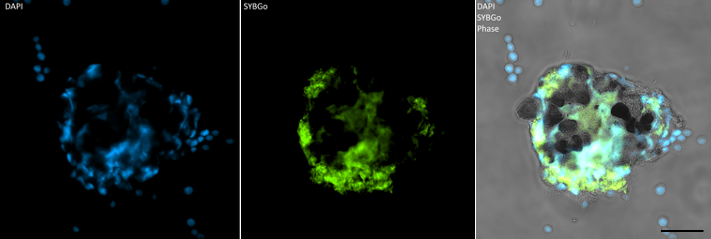


Supplementary Figure 3: **Fluorescent wide-field microscopy of an *ex vivo* granuloma-like structure** displaying insect haemocyte extracellular traps (IHET). Fused haemocytes were identified through individually indistinguishable cellular margins (phase) and nucleus (DAPI, blue), closely resembling neutrophil extracellular trap (NET). Large numbers of SAMTB lux (SYBR gold, green) are associated with the granuloma-like structure, which appears to be localised to the site of IHET. Scale bar represents 50 µm.
