# Supplementary material for: Galleria mellonella as an infection model for the virulent Mycobacterium tuberculosis H37Rv
Source: Virulence. 2022 Sep 11;13(1):1543–57. doi: 10.1080/21505594.2022.2119657 (PMC9481108; doi:10.1080/21505594.2022.2119657)
Supplement: Supplemental Material [file KVIR_A_2119657_SM2614.zip › supplementary/Supplementary Table 1.pdf]

Supplementary Table 1: MIC of antimycobacterial compounds against H37Rv

Antimycobacterial compound	MIC (μg/ml)
INH	0.5
RIF	< 0.125
ETH	2
PZA	> 16
PIP	320

MIC assays were conducted to determine the efficacy of isoniazid (INH), rifampicin (RIF), ethambutol (ETH), pyrazinamide (PZA) and piperacillin (PIP) using the broth dilution method as previously described (1, 2). Compounds we evaluated at a range of 16 to 0.125 μg/ml for INH, RIF, ETH and PZA; and 1280 to 10 μg/ml (PIP) in a two-fold serial dilution step range against a starting inoculum of starting inoculum of 10^7 CFU/ml (OD_{600}: 0.1). MIC was measured 1 week post incubation in a rocking incubator (37 °C and 20 rpm) and were defined as the concentration of compounds required to inhibit growth of mycobacteria, measurable via visual observation and spectrophotometry. MIC was defined through two independent experiments.

1. Asai M, Li Y, Spiropoulos J, Cooley W, Everest D, Robertson BD, Langford PR, Newton SM. 2020. A novel biosafety level 2 compliant tuberculosis infection model using a Δ*leuD*Δ*panCD* double auxotroph of *Mycobacterium tuberculosis* H37Rv and *Galleria mellonella*. Virulence 11:811–824.

2. Asai M, Li Y, Singh Khara J, Robertson BD, Langford PR, Newton SM. 2019. *Galleria mellonella*: an infection model for screening compounds against the *Mycobacterium tuberculosis* complex. Frontiers in Microbiology 10:2630.
